# Disclosure of human immunodeficiency virus status to children in South Africa: A comprehensive analysis

**DOI:** 10.4102/sajhivmed.v20i1.884

**Published:** 2019-08-22

**Authors:** Sabine L. van Elsland, Remco P.H. Peters, Cornelis Grobbelaar, Patiswa Ketelo, Maarten O. Kok, Mark F. Cotton, A. Marceline van Furth

**Affiliations:** 1 Department of Paediatric Infectious Diseases and Immunology, Amsterdam University Medical Center, Vrije Universiteit Amsterdam, Amsterdam, the Netherlands; 2 Department of Paediatrics and Child Health, Tygerberg Children’s Hospital, Stellenbosch University, Cape Town, South Africa; 3 Anova Health Institute, Johannesburg, South Africa; 4 Department of Health Care Governance, Erasmus School of Health Policy and Management, Erasmus University Rotterdam, Rotterdam, the Nertherlands; 5 FAM-CRU, Department of Paediatrics and Child Health, Stellenbosch University, Tygerberg Hospital, Cape Town, South Africa

**Keywords:** Disclosure, Child, Paediatric, HIV, Quality of life, South Africa

## Abstract

**Background:**

The extent of disclosure of HIV status to children and adolescents and the context facilitating their disclosure process have received little attention.

**Objectives:**

To assess disclosure and provide a comprehensive analysis of characteristics associated with disclosure to children (3–14 years) receiving antiretroviral treatment in a South African semi-urban clinic.

**Methods:**

This cross-sectional study used structured interview administered questionnaires which were supplemented with medical record data. Predictors included child, caregiver, clinical and socio-economic characteristics, viral suppression, immune response, adherence, health-related quality of life and family functioning.

**Results:**

We included 190 children of whom 45 (23.7%) received disclosure about their HIV status, of whom 28 (14.7%) were partially disclosed and 17 (8.9%) were fully disclosed. Older age of the child and higher education of the caregiver were strongly associated with disclosure. Female caregivers, detectable viral load, syrup formulation, protease inhibitor (PI) regimens with stavudine and didanosine, and self-reported non-adherence were strongly associated with non-disclosure.

**Conclusion:**

When children do well on treatment, caregivers feel less stringent need to disclose. Well-functioning families, higher educated caregivers and better socio-economic status enabled and promoted disclosure. Non-disclosure can indicate a sub-optimal social structure which could negatively affect adherence and viral suppression. There is an urgent need to address disclosure thoughtfully and proactively in the long-term disease management. For the disclosure process to be beneficial, an enabling supportive context is important, which will provide a great opportunity for future interventions.

## Introduction

Globally, 36.7 million people live with human immunodeficiency virus (HIV), of whom an estimated 2.1 million are children (0–14 years).^1^ Fifteen per cent (320 000) of these children live in South Africa.^1,2^ South Africa has more people receiving antiretroviral therapy (ART) than any other country in the world.^3^ In 2016, the coverage of paediatric ART was 55.0%, reaching 172 000 children.^2^ Depending on measure and definition, paediatric ART adherence ranges between 20.5% and 89.1%.^4^ Poor adherence to medication is common, which contributes to substantial worsening of disease, death and increased healthcare costs.^5^ Factors associated with ART adherence reported in a South African paediatric population include the impact of the condition on daily life, household functioning, socio-economic status (SES), problems administering medication and disclosure.^4^ Non-disclosure of HIV status to the child can lead to a delay in access to treatment, non-adherence and consequent treatment failure.^6,7,8,9^ Although studies have suggested both positive and negative effects of disclosure for children,^10^ the lack of disclosure of HIV status to children and adolescents ultimately adversely affects their well-being.^7^

The availability and roll-out of treatment for adults and children highlight the need to address disclosure.^7^ A review showed that the minority of HIV-infected children in resource-limited settings know their HIV status, and identified child, caregiver, clinical and socio-economic characteristics associated with disclosure.^10^ These predictors are not all studied within the same population. Delaying the initiation of the disclosure process makes it an increasingly difficult process.^9^ Research is needed on effective strategies for disclosure in resource-limited settings.^10,11^ Reported full disclosure to the child in South Africa ranges between 7.9% and 9.0%.^12,13,14^

The South African National Department of Health has committed to prioritise support and guide primary caregivers and healthcare providers for disclosure. This approach intends to ensure the physical, emotional, cognitive and social well-being of the child.^8^ A literature review including 17 studies in low-middle-income countries reported a mean age for disclosure as 9.6 years (8.1–15.0), and that 20.4% of children (3.2% – 69.2%) knew their status.^15^ National guidelines recommend all children from age 3 years to be prepared for disclosure. Disclosure is the first step for children transitioning into adolescents and young adults who successfully manage their own HIV care.^16^

To support the implementation of disclosure guidelines, we assessed the prevalence of disclosure of children’s HIV status to them. In addition, to better understand disclosure, we explored the association between disclosure and child, caregiver, clinical and socio-economic characteristics.

## Methods

This cross-sectional study is a sub-analysis of data published elsewhere, which focused on ART adherence in a population of active paediatric patients aged 2–14 years who were on treatment at TC Newman Clinic – a semi-urban ART clinic in the Western Cape, South Africa – and their caregivers.^4^ For this sub-analysis, we included all children aged 3–14 years who were on treatment between September 2012 and September 2013. The age group was based on national disclosure guidelines.^9^ Children and their caregivers who did not meet these inclusion criteria were excluded from the study. For this study, we assessed prevalence of disclosure and explored all possible characteristics associated with disclosure. Structured questionnaires were administered in interviews while patients were waiting to see the doctor and supplemented with medical record data.

### Definition of disclosure

Paediatric disclosure can refer to disclosure of the child’s HIV status to the child, caregivers’ HIV status to children or children’s disclosure of their own HIV status to others. This study focused on disclosure of the child’s HIV status to the child. Based on caregiver interview, healthcare provider report and medical files, we categorised disclosure status into non-disclosure (the child is unaware of his or her condition and its effect on the body), partial disclosure (the child is aware of his or her condition without naming HIV) and full disclosure (the child is made aware of his or her condition which is named as HIV).^9^ When referred to disclosure, we consider both partial and full disclosure unless otherwise specified.

### Measurements

To provide a comprehensive analysis of predictor variables (child, caregiver, clinical and socio-economic characteristics) and their association with disclosure, we included general demographic information, supplemented with questionnaires. The validated PedsQL^TM^ questionnaires measured health-related quality of life (HRQoL) combining caregiver proxy-report and child self-report (all children ≥ 5 years), and the impact of paediatric chronic health conditions on family and caregivers (family impact).^17,18,19^ Socio-economic status was calculated using 21 questions from the Census 2011.^20^ A higher score (%) indicated better HRQoL, overall family functioning and SES. A combination of adherence monitoring measures was included. Pill count was calculated using the number of pills taken or the volume for liquid formulations (dispensed minus returned) as a percentage of medication prescribed. Adherence was defined as 95% – 105% (a score > 100% could be explained by ingestion of more pills than prescribed and lost pills). Self-reported adherence for the last 3 days was recorded with the validated paediatric AIDS clinical trials group (PACTG) adherence modules.^21^ Adherence was defined as no missed dosages in the last 3 days for self-report. Treatment success was defined by a suppressed viral load (< 50 copies/mL), and immune response defined by CD4 count (> 5 00 cells/mm^3^). This information was retrieved from medical records (6 months before or 3 months after inclusion). Regimen specifications were retrieved from medical records and questionnaires (formulation, prescription, treatment start, progress, complications, difficulties administering medication, side effects).

### Statistical analyses

All analyses were done using IBM SPSS statistics version 25. To describe the association between possible predictor variables and disclosure, univariate logistic regression analyses were conducted presenting odds ratio (OR) and 95% confidence interval (CI) unless otherwise specified. Multivariate analyses are presented when confounding or effect modification was identified for child’s age or caregiver education. Fisher’s exact *p*-value was presented for cell size below 5. Significance was measured at *p* ≤ 0.05.

To describe the relation between multiple possible predictor variables and disclosure, we present a prediction model which was constructed using the forward selection procedure. This method considered all predictors of disclosure by adding the predictor with the lowest *p*-value under 0.05 to the crude model, which was repeated until no additional predictor had a *p*-value < 0.05. The overall percentage correct classified cases and Hosmer and Lemeshow chi-square test with *p*-value for goodness of fit are presented for each model (good fit is indicated by *p*-value > 0.05).

## Ethical considerations

Stellenbosch University’s human research ethics committee approved this study (N11/11/329). In addition, hospital management approved the study in accordance with Provincial Research Policy (40/2009). Written informed consent was obtained from all caregivers and assent from children older than 7 with normal cognitive functioning.

## Results

At the start of the study, 238 active paediatric patients on ART aged 2–14 years attended the clinic. One caregiver refused to participate and 42 patients were missed because caregivers did not visit on the appointment date. With 5 children younger than 3 years of age, this sub-analysis included 190 children. For five households with two children in the study, only the child enrolled first was considered for SES analyses (*n* = 185).

### Disclosure of human immunodeficiency virus status to the child

Most of the children (145 of 190, 76.3%) had not received disclosure about their HIV status, 28 children (14.7%) had received partial disclosure and 17 children (8.9%) had full disclosure. None of the children in early childhood (3–5 years) received disclosure (*n* = 49), 11 of 89 children (12.8%) aged 6–9 years and 34 of 52 (65.4%) young adolescents aged 10–14 years received disclosure. The youngest child disclosed to about their HIV status was 6.6 years and the oldest child who was not disclosed was 12.2 years.

### Child characteristics

Child characteristics associated with disclosure were age and HRQoL. The children were aged 3.2–12.9 years, the majority (74.2%) were of school going age (6 years and older) and 27.4% were young adolescents (10–14 years). Older children (young adolescents) were significantly more likely to be disclosed compared to younger children (under 10 years) (odds ratio [OR] 21.81; 9.41–50.52). Mean self-reported HRQoL index was 91.5%. Children who rated their HRQoL highly were less likely to have received disclosure compared to children who had low HRQoL (OR 0.29; 0.09–0.91). This association attenuated in multivariate analyses (OR 0.58; 0.15–2.30). We did not find significant associations between disclosure and sex of the child, overall HRQoL or school functioning (caregiver proxy-report or self-report) (Table 1).

**TABLE 1:** Associations between disclosure and child characteristics – Univariate analyses.

Child characteristics	Total			Disclosure								
	Mean	*n*	%	Non-disclosed (mean)	Non-disclosed (*n*)	Non-disclosed (%)	Full/partial (mean)	Full/partial (*n*)	Full/partial (%)	Odds Ratio	95% CI	*p*
**Age (*N* = 190)**												
Mean (s.d.)	8.1 (2.6)	-	-	7.3 (2.3)	-	-	10.7 (1.4)	-	-	-	-	-
Median (IQR)	8.5 (5.8-10.2)	-	-	7.3 (5.2-9.0)	-	-	10.8 (9.9-11.8)	-	-	-	-	-
**Age (*N* = 190)**												
3–5 years	-	49	25.8	-	49	33.8	-	0	0	-	-	-
6–9 years	-	89	46.8	-	78	53.8	-	11	24.4	-	-	0.008*†
10–14 years	-	52	27.4	-	18	12.4	-	34	75.6	-	-	0.000*†
**Age (*N* = 190)**												
Young child (< 10 years)	-	138	72.6	-	127	87.6	-	11	24.4	-	-	-
Early adolescence (≥ 10 years)	-	52	27.4	-	18	12.4	-	34	75.6	21.81	9.41–50.52*	0.000*
**Sex (*N* = 190)**												
Female	-	109	57.4	-	84	57.9	-	25	55.6	-	-	-
Male	-	81	42.6	-	61	42.1	-	20	44.4	1.10	0.56-2.16	0.778
**HRQoL - Overall (*N* = 155)**												
Mean (s.d.)	90.5 (10.4)	-	-	90.1 (11.3)	-	-	92.0 (6.4)	-	-	-	-	-
**HRQoL - Overall (*N* = 155)**												
12.8–88.0	-	47	25.1	-	35	24.6	-	12	26.7	-	-	-
88.1–93.0	-	46	24.6	-	39	27.5	-	7	15.6	0.52	0.19–1.48	0.222
93.1–96.6	-	45	24.1	-	30	21.1	-	15	33.3	1.46	0.59–3.60	0.412
96.7–100	-	49	26.2	-	38	26.8	-	11	24.4	0.84	0.33–2.16	0.724
**HRQoL - Self-report (*N* = 155)**												
Mean (s.d.)	91.5 (11.4)	-	-	90.0 (9.1)	-	-	91.9 (12.0)	-	-	-	-	-
**HRQoL - Self-report (*N* = 155)**												
6.5–88.0	-	36	23.2	-	23	19.5	-	13	35.1	-	-	-
88.1–94.5	-	42	27.1	-	33	28.0	-	9	24.3	0.48	0.18–1.32	0.154
94.6–99.9	-	41	26.5	-	31	26.3	-	10	27	0.57	0.21–1.53	0.265
100	-	36	23.2	-	31	26.3	-	5	13.5	0.29	0.09–0.91*	0.035*
**HRQoL school - Self-report (*N* = 147)**												
Mean (s.d.)	82.8 (18.2)	-	-	83.6 (18.1)	-	-	80.5 (18.4)	-	-	-	-	-
**HRQoL school - Self-report (*N* = 147)**												
5.0–74.9	-	34	23.1	-	25	22.7	-	9	24.3	-	-	-
75.0–89.9	-	41	27.9	-	29	26.4	-	12	32.4	1.15	0.42–3.18	0.778
90.0–99.9	-	31	21.1	-	23	20.9	-	8	21.6	0.97	0.32–2.93	0.951
100	-	41	27.9	-	33	30.0	-	8	21.6	0.67	0.23–1.99	0.475
**HRQoL school - Proxy-report (*N* = 172)**												
Mean (s.d.)	81.6 (19.3)	-	-	82.3 (18.9)	-	-	79.3 (20.5)	-	-	-	-	-
**HRQoL school - Proxy-report (*N* = 172)**												
5.0–74.9	-	36	20.9	-	28	20.9	-	8	21.1	-	-	-
75.0–89.9	-	56	32.6	-	39	29.1	-	17	44.7	1.53	0.58–4.03	0.394
90.0–99.9	-	40	23.3	-	35	26.1	-	5	13.2	0.5	0.15–1.70	0.267
100	-	40	23.3	-	32	23.9	-	8	21.1	0.88	0.29–2.64	0.813

CI, confidence interval; s.d., standard deviation; HRQoL, health-related quality of life; IQR, interquartile range.

* , Significant (*p* < 0.05);

† , *p*-value Fisher’s exact test (cell size below 5).

### Caregiver characteristics

Caregiver characteristics associated with disclosure were sex, education and HRQoL. The minority of caregivers were males (7.9%). Young children (under 10 years) of male caregivers were more likely to have received disclosure compared to young children of female caregivers (OR 5.58; 1.24–25.19). Most caregivers had not completed high school education (87.3%). Caregivers who completed their high school education were more likely to disclose the child’s HIV status to the child (multivariate OR 4.04; 1.26–12.91) than those who had not completed their high school education. Caregivers rated their own quality of life index at 90.5% (mean). Caregivers who rated their quality of life higher were less likely to disclose the child’s HIV status to the child (OR 0.31; 0.10–0.95). This association attenuated in multivariate analyses (OR 0.64; 0.16–2.54). We did not find significant associations between disclosure and caregiver’s age, relationship with the child, cultural background, caregiver’s marital status or worry as indicators of caregiver functioning (extent of concern about chil d’s treatment, side effects, reaction of others, child’s condition or effects of illness on family and future) (Table 2).

**TABLE 2:** Associations between disclosure and caregiver characteristics – Univariate analyses.

Caregiver characteristics	Total			Disclosure								
	Mean	*n*	%	Non-disclosed (mean)	Non-disclosed (*n*)	Non-disclosed (%)	Full/partial (mean)	Full/partial (*n*)	Full/partial (%)	Odds Ratio	95% CI	*p*
**Age (*N* = 190)**												
Mean (s.d.)	39.2 (11.2)	-	-	38.6 (11.2)	-	-	40.8 (11.0)	-	-	-	-	-
Median (IQR)	37.3 (31.7-44.1)	-	-	36.1 (31.1-44.0)	-	-	39.0 (34.5-47.2)	-	-	-	-	-
**Age (*N* = 190)**												
16.0-31.6	-	47	24.7	-	39	26.9	-	8	17.8	-	-	-
31.7-37.2	-	47	24.7	-	39	26.9	-	8	17.8	1.00	0.34-2.93	1.000
37.3-44.5	-	49	25.8	-	32	22.1	-	17	37.8	2.59	0.99-6.78	0.052
44.6-74.5	-	47	25.8	-	35	24.1	-	12	26.7	1.67	0.61-4.56	0.316
**Sex (*N* = 190)**												
Female	-	175	92.1	-	134‡	93.7‡	-	41‡	72.7‡	-	-	-
Male	-	15	7.9	-	11‡	6.3‡	-	4‡	27.3‡	5.58‡	1.24-25.19*‡	0.025*‡
**Relation to child (*N* = 190)**												
Parent	-	132	69.5	-	103	71.0	-	29	64.4	-	-	-
Other	-	58	30.5	-	42	29.0	-	16	35.6	1.35	0.67-2.75	0.403
**Language (*N* = 190)**												
Afrikaans	-	56	29.5	-	43	29.7	-	13	28.9	-	-	-
Xhosa	-	127	66.8	-	98	67.6	-	29	64.4	0.98	0.46-2.06	0.955
Other	-	7	3.7	-	4	2.8	-	3	6.7	2.48	0.49-12.54	0.272
**Marital Status (*N* = 190)**												
Not Married	-	135	71.1	-	108	74.5	-	27	60.0	-	-	-
Married	-	55	28.9	-	37	25.5	-	18	40.0	1.95	0.96-3.93	0.064
**Education (*N* = 189)**												
Primary school	-	165	87.3	-	131	91.0	-	34	75.6	-	-	-
High school	-	24	12.7	-	13	9.0	-	11	24.4	3.26	1.34-7.92*	0.009*
**HRQoL (*N* = 181)**												
Mean (s.d.)	90.5 (12.2)	-	-	90.5 (12.3)	-	-	90.6 (12.3)	-	-	-	-	-
**HRQoL (*N* = 181)**												
36.3-84.3	-	47	26.0	-	33	23.9	-	14	32.6	-	-	-
87.4-94.6	-	43	23.8	-	38	27.5	-	5	11.6	0.31	0.10-0.95*	0.041*
94.7-99.9	-	47	26.0	-	35	25.4	-	12	27.9	0.81	0.33-2.00	0.645
100	-	44	24.3	-	32	23.2	-	12	27.9	0.88	0.36-2.12	0.791
**FI Worry (*N* = 188)**												
Mean (s.d.)	89.2 (11.4)	-	-	89.6 (10.9)	-	-	88.0 (13.0)	-	-	-	-	-
**FI Worry (*N* = 188)**												
50.0-84.9	-	45	23.9	-	33	22.9	-	12	27.3	-	-	-
85.0-94.9	-	58	30.9	-	47	32.6	-	11	25.0	0.64	0.25-1.63	0.354
95.0-99.9	-	23	12.2	-	17	11.8	-	6	13.6	0.98	0.31-3.04	0.959
100	-	62	33.0	-	47	32.6	-	15	34.1	0.88	0.36-2.12	0.771

CI, confidence interval; s.d., standard deviation; HRQoL, health-related quality of life; FI, Family impact; IQR, interquartile range.

* , Significant (*p* < 0.05);

‡ , presented for children under 10 years.

### Clinical characteristics

Clinical characteristics associated with disclosure included suppressed viral load, formulation (tablet/syrup), non-nucleoside reverse transcriptase inhibitors (NNRTI) in regimen, protease inhibitor (PI) in regimen with stavudine and didanosine, regimens with efavirenz, longer duration on treatment, start of treatment in the first year of life, experiencing difficulties administering treatment and poor adherence to treatment. One-third (32.8%) of children had a detectable viral load and had less likely received disclosure compared to those with a suppressed viral load (multivariate OR 0.21; 0.05–0.84). Most children were on a regimen with a combination of three medicines (86.3%), consisting of tablets only (62.2%). Children whose regimen included syrups (syrups only or combined with tablets) had less likely received disclosure compared to children who were on tablets only (multivariate OR 0.28; 0.08–0.92).

Children on a regimen including an NNRTI (35.3%) more likely received disclosure compared to children on a regimen with no NNRTIs (OR 2.71; 1.37–5.38). This association attenuated in multivariate analyses (OR 1.84; 0.78–4.31). Children on a PI-based regimen with stavudine and didanosine (16.8%) less likely received disclosure compared to children who were on a non-PI-based regimen (multivariate OR 0.19; 0.03–1.00). Children on a regimen including efavirenz more likely received disclosure than those with no efavirenz (OR 2.90; 1.46–5.77). This association attenuated in multivariate analyses (OR 1.91; 0.81–4.48). Children on a regimen of lopinavir/ritonavir syrup (79.5%) less likely received disclosure (OR 0.14; 0.03–0.59). This association attenuated in multivariate analyses (OR 0.54; 0.11–2.62). Children were on treatment for 1 month to 9.8 years (mean 5.2 years). Children with a longer treatment duration more likely received disclosure compared to those more recently initiating treatment (OR3.02; 1.19–7.63). This association attenuated in multivariate analyses (OR 1.21; 0.38–3.91). Children who started their treatment in the first year of their life (30.5%) less likely received disclosure than those commencing treatment later in life (OR 0.12; 0.04–0.40). This association attenuated in multivariate analyses (OR 0.49; 0.12–1.94). Caregivers who experienced difficulties administering medication (30.5%) less likely disclosed the child’s HIV status to the child compared to caregivers not experiencing difficulties administering medication (OR 0.41; 0.18–0.95). This association attenuated in multivariate analyses (OR 0.63; 0.23–1.73). Non-adherence was 10.1% for self-report and 63.1% for pill count. Children who were non-adherent to their treatment had less likely received disclosure than those who were adherent (self-report Fisher’s exact *p*-value 0.008). We did not find any significant associations between disclosure and WHO clinical staging, CD4 count, complications reported (e.g. running out of medication, flavour, forgetting, multiple caregivers, illness, depression and being away from home), side effects (e.g. fever, rash, sleep disturbance and pain), default on treatment in the past and subsequently restarted, number of medicines in regimen or adherence defined by pill count (95% – 105%) (Table 3).

**TABLE 3:** Associations between disclosure and clinical characteristics – Univariate analyses.

Clinical characteristics	Total			Disclosure								
	Mean	*n*	%	Non-disclosed (mean)	Non-disclosed (*n*)	Non-disclosed (%)	Full/partial (mean)	Full/partial (*n*)	Full/partial (%)	Odds Ratio	95% CI	*p*
**WHO clinical (*N* = 184)**												
Stage 1	-	19	10.3	-	14	10.1	-	5	11.1	-	-	-
Stage 2	-	48	26.1	-	34	24.5	-	14	31.1	1.15	0.353.81	0.816
Stage 3	-	83	45.1	-	64	46.0	-	19	42.2	0.83	0.27-2.61	0.751
Stage 4	-	34	18.5	-	27	19.4	-	7	15.6	0.73	0.20-2.71	0.634
**Health Outcome VL (*N* = 125)**												
Suppressed VL	-	84	67.2	-	59	62.1	-	25	83.3	-	-	-
Detectable VL	-	41	32.8	-	36	37.9	-	5	16.7	0.33	0.12-0.93*	0.037*
**Health Outcome CD4 (*N* = 118)**												
CD4 count ≥ 500 cells/mm^3^	-	109	92.4	-	86	93.5	-	23	88.5	-	-	-
CD4 count < 500 cells/mm^3^	-	9	7.6	-	6	6.5	-	19	11.5	1.87	0.43-8.05	0.401
**Complicatoins (*N* = 182)**												
No	-	151	83.0	-	115	82.7	-	36	83.7	-	-	-
Yes	-	31	17.0	-	24	17.3	-	7	16.3	0.93	0.37-2.34	0.88
**Difficulties (*N* = 187)**												
No	-	130	69.5	-	93	65.5	-	37	82.2	-	-	-
Yes	-	57	30.5	-	49	34.5	-	8	17.8	0.41	0.18-0.95*	0.037*
**Side effects (*N* = 181)**												
No	-	160	88.4	-	122	88.4	-	38	88.4	-	-	-
Yes	-	21	11.6	-	16	11.6	-	5	11.6	1.00	0.35-2.92	0.995
**Treatment duration (*N* = 190)**												
Mean (s.d.)	5.2 (2.4)	-	-	4.9 (2.3)	-	-	6.1 (2.5)	-	-	-	-	-
**Treatment duration (*N* = 190)**												
0.0-3.4	-	49	25.8	-	40	27.6	-	9.0	20.0	-	-	-
3.5-5.5	-	46	24.2	-	40	27.6	-	6.0	13.3	0.67	0.22-2.05	0.479
5.6-6.6	-	48	25.3	-	37	25.5	-	11	24.4	1.32	0.49-3.55	0.580
6.7-9.9	-	47	24.7	-	28	19.3	-	19	42.2	3.02	1.19-7.63*	0.020*
**Treatment 1st life year (*N* = 190)**												
No	-	132	69.5	-	90	62.1	-	42	93.3	-	-	-
Yes	-	58	30.5	-	55	37.9	-	3	6.7	0.12	0.04-0.40*	0.001*
**Treatment interrupted (*N* = 188)**												
No	-	173	92	-	130	90.9	-	43	95.6	-	-	-
Yes	-	15	8	-	13	9.1	-	2	4.4	0.47	0.10-2.14	0.326
**Regimen (*N* = 190)**												
Standard 3 meds	-	164	86.3	-	124	85.5	-	40	88.9	-	-	-
Less (1 or 2)	-	24	12.6	-	19	13.1	-	5	11.1	-	-	0.803†
More (4 meds)	-	2	1.1	-	2	1.4	-	0	0	-	-	1.000†
**Regiment formulation (*N* = 188)**												
Tablets only	-	117	62.2	-	76	53.1	-	41	91.1	-	-	-
Syrups	-	71	37.8	-	67	46.9	-	4	8.9	0.11	0.04-0.33*	0.000*
**Regimen NNRTI (*N* = 190)**												
No NNRTI	-	123	64.7	-	102	70.3	-	21	46.7	-	-	-
NNRTI	-	67	35.3	-	43	29.7	-	24	53.3	2.71	1.37-5.38*	0.004*
**Regimen PI (*N* = 190)**												
No PI base	-	80	42.1	-	55	37.9	-	25	55.6	-	-	-
PI + D4T/DDI	-	32	16.8	-	30	20.7	-	2	4.4	0.15	0.03-0.66*	0.013*
PI + ABC/AZT	-	63	33.2	-	51	35.2	-	12	26.7	0.52	0.24-1.14	0.101
PI + other	-	15	7.9	-	9	6.2	-	6	13.3	1.47	0.47-4.57	0.509
**Regimen EFV (*N* = 190)**												
No EFV	-	125	65.8	-	104	71.7	-	21	46.7	-	-	-
EF	-	65	34.2	-	41	28.3	-	24	53.3	2.90	1.46-5.77*	0.002*
**Regimen lop/rit (*N* = 190)**												
No lop/rit syrup	-	151	79.5	-	108	74.5	-	43	95.6	-	-	-
Lop/rit syrup	-	39	20.5	-	37	25.5	-	2	4.4	0.14	0.03-0.59*	0.008*
**Adherence 3-day self-report (*N* = 188)**												
Adherent	-	169	89.9	-	124	86.7	-	45	100	-	-	-
Non-adherent	-	19	10.1	-	19	13.3	-	0	0	-	-	0.008*†
**Adherence pill count 95%-105% (*N* = 187)**												
Adherent	-	69	36.9	-	48	33.6	-	21	47.7	-	-	-
Non-adherent	-	118	63.1	-	95	66.4	-	23	52.3	0.55	0.28-1.10	0.091

CI, confidence interval; s.d., standard deviation; NRTI, nucleoside reverse transcriptase inhibitors; NNRTI, non-nucleoside reverse transcriptase inhibitors; PI, protease inhibitor; D4T,

stavudine; DDI, didanosine; ABC, abacavir; AZT, zidovudine; EFV, efavirenz; lop/rit, lopinavir/ritonavir.

* , Significant (*p* < 0.05),

† , *p*-value Fisher’s exact test (cell size below 5).

### Socio-economic characteristics

Socio-economic characteristics associated with disclosure included family functioning, affected daily activities and waterborne sanitation. Overall family impact index was 90.4% (mean). Children with a high overall family impact scale (good family functioning) had more likely received disclosure than those from a household with low family impact index (OR 4.18; 1.54–11.32). This association attenuated in multivariate analyses (OR 0.80; 0.22–3.00). The mean score for daily activity index (component of family functioning) was 91.5% and included the extent of activities taking more time and effort, difficulty finding time and energy to finish household tasks or affected daily activities. Children from families with a higher family activity index had less likely received disclosure compared to children from families with a low family activity index (activities affected) (OR 0.21; 0.04–1.000). This association attenuated in multivariate analyses (OR 0.81; 0.30–2.17). The overall mean SES index was 52.0%. The study population had significantly more often waterborne sanitation (73.7%, *p* < 0.001), owned a TV (89.4%, *p* < 0.001), fridge (79.9%, *p* = 0.001) or cell phone (95.2%, *p* = 0.003) than the general South African population. However, the study population lived with significantly more people in one household (mean 5.2, *p* < 0.001), more people lived in informal dwellings (39.5%, *p* < 0.001) and were less likely to own a computer (11.5%, *p* = 0.001), landline phone (7.1%, *p* = 0.004) or car (15.3%, *p* < 0.001) compared to the general South African population (Table 4). Children from households with waterborne sanitation had more likely received disclosure than those from households with no toilet facilities connected to sewage (OR 2.87; 1.13–7.29). This association attenuated in multivariate analyses (OR 1.76; 0.58–5.35). We did not find any significant associations between disclosure and overall SES index (Table 5).

**TABLE 4:** Associations between disclosure and socio-economic characteristics – Univariate analyses.

Socio-economic characteristics	Total			Disclosure								
	Mean	*n*	%	Non-disclosed (mean)	Non-disclosed (*n*)	Non-disclosed (%)	Full/partial (mean)	Full/partial (*n*)	Full/partial (%)	Odds Ratio	95% CI	*p*
**FI Overall (*N* = 189)**												
Mean (s.d.)	90.4 (11.5)			89.7 (10.8)			92.4 (13.2)			-	-	-
**FI Overall (*N* = 189)**												
41.9-87.4	-	47	24.9	-	40	27.8	-	7	15.6	-	-	-
97.5-93.3	-	47	24.9	-	38	26.4	-	9	20.0	1.35	0.46-4.00	0.584
93.4-99.1	-	50	26.5	-	40	27.8	-	10	22.2	1.43	0.50-4.13	0.510
99.2-100	-	45	23.8	-	26	18.1	-	19	42.2	4.18	1.54-11.32*	0.005*
**FI Activities (*N* = 189)**												
Mean (s.d.)	91.4 (15.2)		-	91.3 (15.2)		-	91.5 (15.4)		-	-	-	
**FI Activities (*N* = 189)**												
25.0-91.6	-	44	23.3	-	32	22.2	-	12	26.7	-	-	
91.7	-	28	14.8	-	26	18.1	-	2	4.4	0.21	0.04-1.000*	0.050*
100	-	117	61.9	-	86	597	-	31	68.9	0.96	0.44-2.10	0.921
**SES-Index (*N* = 183)**												
Mean (s.d.)	52.0 (17.0)		-	50.7 (17.2)		-	56.4 (15.8)		-	-	-	-
**SES-Index (*N* = 183)**												
9.5-42.7	-	40	21.9	-	34	24.3	-	6	14.0	-	-	-
42.8-57.0	-	47	25.7	-	36	25.7	-	11	25.6	1.73	0.58-5.20	0.328
57.1-66.6	-	44	24.0	-	34	24.3	-	10	23.3	1.67	0.55-5.10	0.371
66.7-100	-	52	28.4	-	36	25.7	-	16	37.2	2.52	0.88-7.19	0.084
**SES Toilet facility (*N* = 185)**												
No sewage	-	50	27.0	-	44	31.2	-	6	13.6	-	-	-
Water-born	-	135	73.0	-	97	68.8	-	38	86.4	2.87	1.13-7.29*	0.026*

CI, confidence interval; s.d., standard deviation; FI, Family impact; SES, socio-economic status.

* , Significant (*p* < 0.05).

**TABLE 5:** Socio-economic status indicators and South African comparison.

Variable	*N*	Study (%)	South Africa† (%)	Chi-squared	*p*
Number of people per household	184	5.2	3.4	*t* = 11.3	0.000*
Type of dwelling (formal / informal)	185	60.0	77.6	32.0	0.000*
Drinking water (piped in house or yard/other)	185	77.8	73.4	1.8	0.176
Toilet facilities (waterborne /no sewage)	185	73.0	57.0	19.3	0.000*
Share toilet facilities (no/yes)	183	52.5	-		-
Fuel cooking (electricity/other)	185	77.3	73.9	1.1	0.109
Fuel heating (electricity/other)	185	58.9	58.8	0.0	0.978
Fuel lighting (electricity/other)	185	83.2	84.7	0.3	0.571
Material floor (finished/natural or rudimentary)	185	95.7	-	-	-
Material walls (finished/unfinished)	185	39.5	-	-	-
Share rooms in house (no/yes)	183	82.0	-	-	-
Radio (no/yes)	184	72.8	67.5	2.4	0.125
TV (no/yes)	184	89.1	74.5	20.6	0.000*
Fridge (no/yes)	184	79.9	68.4	11.3	0.001*
Computer (no/yes)	184	11.4	21.4	10.9	0.001*
Landline (no/yes)	184	7.1	14.5	8.1	0.004*
Cell phone (no/yes)	184	95.7	88.9	8.6	0.003*
Car (no/yes)	184	15.8	29.5	16.6	0.000*
Bicycle (no/yes)	184	16.3	-	-	-
Motorcycle/Scooter (no/yes)	184	1.1	-	-	-
Donkey/horse (no/yes)	184	0	-	-	-
Sheep/cattle/goat (no/yes)	184	0	-	-	-

* , Significant (*p* < 0.05),

† , StatsSA 2012.^20^

### Prediction model

**FIGURE 1:**
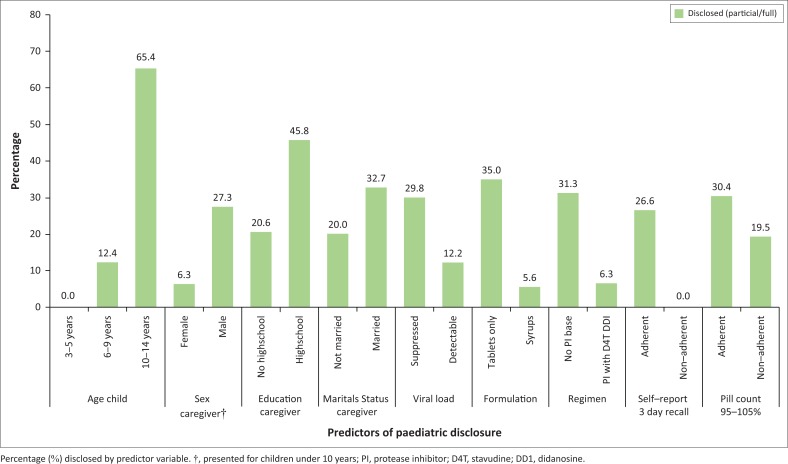
Predictors of paediatric disclosure.

The prediction model for disclosure included five variables: age of the child (OR 146.56; 20.27–1059.69, *p* < 0.001), PI regimen with stavudine and didanosine (OR 0.01; 0.00–0.22, *p* = 0.005), marital status (OR 7.00; 1.39–35.03, *p* = 0.018), viral load (OR 0.05; 0.01–0.41, *p* = 0.005) and adherence (pill count 95% – 105%) (OR 0.16; 0.03–0.77, *p* = 0.023). The association with caregiver education attenuated when adding viral load to the model. The overall percentage of correctly classified cases was 91.1% and Hosmer and Lemeshow’s chi-square test for goodness of fit was 58.7 (*p* = 0.812). Figure 1 provides an overview of the proportion who received children disclosure within the categories of all predictors identified in multivariate analyses and the prediction model.

## Discussion

Only 17 children (8.9%) in this cohort of 3–14-year-olds received full disclosure. In multivariate analyses, we found that increased age of the child and higher education of the caregiver were strongly associated with disclosure of HIV status to the child. In addition, sex of the caregiver, detectable viral load, syrup formulation, PI regimens with stavudine and didanosine, and self-reported non-adherence were strongly associated with non-disclosure. The prediction model identified age of the child, caregiver’s marital status, viral load, regimen and non-adherence defined by pill count (95% – 105%) as predictors of disclosure.

Similar to other studies, we found older age of the child to be strongly associated with increased probability of disclosure of the HIV status to the child.^14,22,23^ Literature does not specifically associate better HRQoL of the child with non-disclosure; however, health-related factors and a child’s family situation are reported as predictors of disclosure.^10^

Male caregiver, level of education and HRQoL were associated with disclosure. While some studies have described not having a biological father as a predictor of disclosure,^24,25^ we found that children had more likely received disclosure when their main caregiver was their father. Both the events of the demise of one’s father and the absence of the mother in the household indicate major life events that are possibly related to HIV. This could explain the association between caregiver’s gender and disclosure, as disclosure is more likely to happen when the caregivers themselves are HIV-positive.^26^ While some studies have confirmed our finding that caregivers with higher education are more likely to disclose the child’s HIV status to their child,^27^ other studies have not.^14^ Caregivers feeling worried and unprepared for the process of disclosure and answering questions prevent actual disclosure.^22,28^ The association we found between educational level and disclosure might be explained by better educated caregivers feeling more equipped to start this process. Our finding that caregivers with better HRQoL are a predictor of non-disclosure is not reported in other literature, although the child’s family situation and caregiver disclosure-related anxiety are described to affect disclosure.^28^

We found a strong association between detectable viral load and non-disclosure. A detectable viral load is an indicator of failure of treatment.^29^ Conversely, addressing disclosure could positively affect adherence and viral suppression.^6,23^ Non-adherence was associated with non-disclosure. Most likely, this association was reversed where non-disclosure contributed to difficulties remaining adherent.^4^ Similarly, caregivers experiencing difficulties administering medication had less likely disclosed the child’s HIV status. Non-disclosure may have contributed to difficulties administering medication. We did not find an association between CD4 count and disclosure. Some literature described that children with a CD4 percentage over 15% are more likely to receive disclosure,^24^ where others did not confirm this association for CD4 percentage or CD4 count.^14^ Children on regimens including syrups were less likely to receive disclosure. Although young children were generally on syrup formulations, the association remained when corrected for age. Possibly an easier routine with syrups does not require the need to disclose. Children on PI-based regimens with stavudine and didanosine had less likely received disclosure. Current guidelines recommend replacing stavudine and didanosine with abacavir^29^ and will therefore not be part of future regimens. Multiple clinical characteristics associated with disclosure in univariate analyses attenuated in multivariate analyses, explained by the child’s age (lopinavir/ritonavir syrup, NNRTI-based regimens, efavirenz regimens, duration on treatment and starting treatment in the first year of life). Children on lopinavir/ritonavir syrup were less likely disclosed. This could be explained by the regimen generally being given to young children and being changed to tablet form for older children. Although the general experience of side effects did not affect disclosure, side effects affecting the central nervous system, unusual dreams and trouble sleeping (efavirenz) and severe rash (nevirapine)^29^ likely contributed to the decision of caretakers to disclose the HIV status to children on regimens including NNRTIs. Children who were on treatment for longer duration had more likely received disclosure. Guidelines in South Africa regard all HIV-positive children eligible to initiate ART irrespective of CD4 count or clinical staging.^29^ Older children, who are more likely on treatment for longer duration, more often receive disclosure,^10^ potentially explaining why the association attenuated in multivariate analysis. Other studies have confirmed the association we find between longer time on ART and disclosure.^25^ Children who started treatment in the first year of life, however, less likely received disclosure. Disclosure did not seem as urgent for caregivers when the same routine with their child could be maintained from birth and no failure of treatment occurred.

Socio-economic characteristics associated with disclosure included family functioning, affected daily activities and waterborne sanitation. Although some studies have described an association with disclosure and the child’s family situation,^10,14^ no specific measures for family functioning or activities were reported in the literature. Indicators of SES including financial problems^24^ and the child being hungry^14^ are reported in the literature as a predictor of disclosure. Although we did not find an association between SES index and disclosure, despite a large number of people living in informal settlements, we found that children from households with access to waterborne sanitation had more likely received disclosure. Informal living conditions more often lack waterborne sanitation, are more densely populated and lack privacy required to support the disclosure process.

A limitation of our study was the reliance on medical records for viral load and CD4 count results. In addition, the questionnaire did not include topics like experience with or perspectives on disclosure. Literature focuses on healthcare providers’ perspective^30,31,32^ or caregivers’ perspective.^12,14,22,33^ The child’s perspective on disclosure is rarely or not studied at all. A strength of our study was that our interviews included all children aged 5 years or older when addressing their HRQoL. Although we suggest doing similar research in other settings to ensure generalisability of the data, another strength of this study was the reasonable sample size.

## Conclusion

This cross-sectional study shows a low proportion of children knowing about their HIV status. Older age of the child was strongly associated with disclosure. We found a less stringent need for caregivers to disclose the child’s HIV status to the child when ART was tolerated well and no condition-related difficulties were experienced (e.g. high HRQoL for both the child and the caregiver and family activities not affected by chronic disease). Well-functioning families, with caregivers who received higher level of education and children from households with better SES, provided an environment enabling and promoting disclosure of the HIV status to the child. Disclosure can only be beneficial when there is a supportive social structure. Non-disclosure can indicate a sub-optimal social structure, which could negatively affect adherence and viral suppression. In order to successfully address disclosure, the complex social context needs to be taken into account. When families are in a good space, there is no pressing need to start the disclosure process. However, these circumstances positively enable the disclosure process. Targeting these families for disclosure interventions and the support of families to reach such an enabling environment can therefore be especially successful.
